# Circulating Neuronal Exosome Cargo as Biomarkers of Neuroplasticity in Cushing’s Syndrome

**DOI:** 10.1007/s12035-025-05069-z

**Published:** 2025-05-24

**Authors:** Marica Pagliarini, Loretta Guidi, Caterina Ciacci, Roberta Saltarelli, Monia Orciani, Marianna Martino, Maria Cristina Albertini, Giorgio Arnaldi, Patrizia Ambrogini

**Affiliations:** 1 Department of Biomolecular Sciences, University of Urbino Carlo Bo, 61029 Urbino, Italy; 2 Department of Neurology, Ulm University, 89081 Ulm, Germany; 3 Department of Clinical and Molecular Sciences-Histology, School of Medicine, University “Politecnica Delle Marche”, 60126 Ancona, Italy; 4 Department of Clinical and Molecular Sciences-Division of Endocrinology and Metabolic Diseases, (DISCLIMO), University “Politecnica Delle Marche”, 60126 Ancona, Italy; 5 Department of Health Promotion, Mother and Child Care, Internal Medicine and Medical Specialties “G. D’Alessandro” (PROMISE), University of Palermo, 90127 Palermo, Italy

**Keywords:** Adult neurogenesis, Glucocorticoids, MicroRNAs, Neuronal exosomes, Circulating biomarkers

## Abstract

The hippocampus is the main target of glucocorticoids (GCs) in the brain since it contains the greatest concentration of the specific receptors. GCs are among the factors modulating adult hippocampal neurogenesis (AHN), which occurs in mammalians, including humans. Prolonged exposure to high GC levels triggers AHN impairment and induces affective and cognitive deficits, consistently with hippocampal neurogenesis functions. Cushing’s syndrome (CS) is a rare endocrine disorder characterized by persistently elevated GC levels, namely, cortisol, that also results in affective disorders and impairment of hippocampus-associated memory, suggesting a disruption of hippocampal neurogenesis. Players of adult neurogenesis process, such as Neural Stem/Progenitor Cells and differentiating neuronal cells, release exosomes able to cross brain blood barrier, reaching the peripheral blood. MicroRNAs are known to be selectively enriched in neuronal exosomes and to play a crucial role in adult neurogenesis regulation. The main question addressed in this exploratory study was whether neuroplasticity-related microRNAs (miRNAs), carried by neuronal-derived exosomes in peripheral blood, could reflect alterations in neurogenic processes associated with Cushing’s syndrome. Hence, in the present work, we measured the content in selected miRNAs of neuronally derived exosomes in peripheral blood of patients affected by endogenous and active CS and age and sex-matched healthy subjects. The human miRNAs (miR-126, miR-9, miR-223, miR-34a, miR-124a, and miR-146a) were quantified by RT-qPCR. All the miRNAs analyzed were significantly differentially expressed in CS patients as compared to healthy subjects. Our findings support the following: (i) patients with Cushing’s syndrome (CS) may exhibit a putative dysregulation of neurogenesis that could underlie the early-onset impairment of affective and cognitive functions; (ii) the exosomal cargo may represent a potential biomarker for monitoring functional and dysfunctional neuroplasticity processes in adult humans. Additional studies are needed to confirm and expand upon the findings across a wider cohort of patients.

## Introduction

Adaptive and maladaptive changes of the nervous system in response to external or internal events are referred to as neuroplasticity processes. These changes are related to structural and functional modifications within neural circuits and have great relevance under physiological and pathological conditions.

Adult neurogenesis is a fascinating phenomenon of neuroplasticity occurring in specific brain regions, among which hippocampal dentate gyrus (DG). Here, new granule neurons are generated from Neural Stem/Progenitor Cells (NSPCs) through a sequential multi-step process and integrated into the existing network [1, 2]. The precise contribution of adult-born neurons to the hippocampal function is still an open question; however, it is generally recognized that they facilitate a variety of cognitive functions, including the long-term consolidation of new hippocampal memories, as well as the forgetting of previously acquired ones, and they are also involved in affective regulation [3]. Although it is mostly accepted that the DG of the hippocampus is evolutionarily preserved as one of the few sites of neurogenesis in adult mammals, evidence on the continuation of the hippocampal neurogenesis in the adult and aging human brain remains ambiguous, mainly due to the methodological difficulties of studying neurogenesis in humans [4, 5].

The neurogenic cascade that leads to new neurons production critically depends on many intrinsic/extrinsic factors (such as aging, environment, hormones, neurochemicals, behavior) and to numerous brain diseases ranging from depression to epilepsy [6–8]. Among the factors affecting adult hippocampal neurogenesis (AHN), glucocorticoids (GCs), adrenal steroid hormones involved in the stress response, have been extensively investigated and a cross-talk between the Hypothalamic–Pituitary–Adrenal axis (HPA) and the AHN has been pointed out [9].

Glucocorticoids act by binding two types of receptors, glucocorticoid receptors (GRs) and mineralocorticoid receptors (MRs), which show differences in their affinity for GCs. Both GRs and MRs are abundant in the DG [10–12] and are differentially expressed in sequential stages of the neurogenic cascade [13]. Since the early 2000 s, animal studies have been demonstrating that GCs/chronic stress/neuroinflammation are among the most important negative regulators of AHN [14, 15] and that GC-mediated disruption of neurogenesis in adults contributes to the occurrence of brain diseases, including cognitive and affective disorders, and neurodegenerative diseases [9].

Cushing’s syndrome (CS) is a rare clinical condition due to overproduction of GCs leading to abnormal and prolonged exposure to cortisol. This complex disease is associated with cardiovascular, metabolic, immunological, neurocognitive, hematological, and infectious complication causing high morbidity and mortality despite treatment [16]. Namely, these patients commonly show affective disorders and impairment of hippocampus-associated memory [17, 18] which are among the earliest symptoms described in CS [19]. Along this line of evidence, several investigations have also revealed hippocampal atrophy in patients with CS [19–23].

Considering the above findings, an impairment of hippocampal neurogenesis, which is involved in learning and memory processes and in affective functions, may be assumed in patients affected by CS. However, as mentioned before, investigating AHN in vivo in humans is not easy to perform as sparse developments have been made regarding specific in vivo measurements using neuroimaging techniques [24], which are also expensive. Therefore, the identification of circulating biomarkers of neuroplasticity might work around this problem, providing evidence on the occurrence of adult neurogenesis process also in humans.

In this context, neuronal exosomes could be of some significance. As a matter of fact, neurons are able to release exosomes, which can cross the blood brain barrier (BBB) [25, 26] reaching the peripheral blood. Exosomes can also derive from NSPCs and differentiating neuronal cells [27] and they have been implicated in all stages of adult neurogenesis [28].

A complex mixture of molecules can be contained in exosomes depending on the cells they are derived from, including microRNAs (miRNAs) [29], which are selectively enriched into exosomes [30–32]. MiRNAs, a class of non-coding small RNA molecules, are important factors for post-transcriptional regulation of gene expression levels [33] and play important roles both in brain development and in adult neurogenesis processes [34]. Altered expression and dysregulation of miRNAs have been linked to pathological states [35].

Since neuronal-derived exosomes can cross the BBB [25, 26], are detectable in plasma, and their molecular cargo likely reflects brain-specific biomarker expression, this exploratory study aimed to investigate whether certain neuroplasticity-related microRNAs within these exosomes could indicate alterations in neurogenic processes associated with Cushing’s syndrome (CS). To this end, we quantified selected miRNAs in neuronal-derived exosomes isolated from the peripheral blood of patients with CS and matched healthy controls, with the goal of gaining insight into neuroplasticity mechanisms—such as neurogenesis—that are implicated in the early cognitive and affective impairments observed in CS. Discovering biomarkers for cognitive impairment in CS could help to clarify the underlying pathogenic processes and improve CS diagnosis, management, and prognosis. In addition, and more generally, the identification of specific exosomal cargoes could serve as a potential biomarker to monitor functional and dysfunctional neuroplasticity processes, such as neurogenesis, in adult humans.

## Materials and Methods

### Participants

Blood samples were collected from 20 patients (7 males and 13 females, average age 43.27 ± 12.8 years) with a confirmed diagnosis of endogenous Cushing’s syndrome (CS). The diagnosis of CS and subsequent etiological diagnosis were made in accordance with the criteria established by the Endocrine Society Guidelines and the 2003 Consensus Statement on the diagnosis and complications of Cushing’s syndrome [16]. Patients with subclinical hypercortisolism, either unilateral or bilateral adrenal enlargement, a history of cortisol-secreting adrenal carcinoma, and Nelson’s Syndrome were excluded.

The CS cohort included patients with hypercortisolism due to either Cushing’s disease (ACTH-secreting pituitary adenoma) or cortisol-secreting adrenal adenoma (Table 1). The former were patients with persistent or recurrent hypercortisolism after pituitary surgery or during medical treatment to control hypercortisolism.

**Table 1:** Clinical data of CS patients

Case	Age	Sex	Diagnosis	Obesity/overweight	Hypertension	Diabetes/IGT	Dyslipidemia	Hypogonadism	Smoke
1	31	F	CD	Yes	Yes	No	No	No	No
2	45	M	CD	Yes	Yes	Yes	Yes	Yes	No
3	24	F	CD	No	Yes	No	No	Yes	No
4	64	F	CSA	Yes	Yes	Yes	Yes	Yes	No
5	44	M	CSA	No	Yes	Yes	Yes	Yes	Yes
6	47	M	CD	Yes	Yes	Yes	Yes	Yes	No
7	63	F	CSA	Yes	Yes	Yes	Yes	Yes	No
8	23	F	CD	No	No	No	Yes	Yes	No
9	38	F	CD	Yes	Yes	Yes	No	Yes	Yes
10	45	M	CD	Yes	Yes	Yes	Yes	No	No
11	53	M	CD	Yes	Yes	Yes	Yes	Yes	No
12	34	F	CSA	Yes	Yes	No	No	Yes	No
13	58	F	CSA	Yes	Yes	Yes	Yes	Yes	Yes
14	25	M	CD	No	No	No	No	No	No
15	35	F	CD	No	Yes	No	No	Yes	No
16	46	F	CD	Yes	Yes	Yes	Yes	Yes	No
17	61	F	CSA	Yes	Yes	Yes	Yes	Yes	No
18	56	M	CD	Yes	Yes	Yes	Yes	Yes	Yes
19	44	F	CD	Yes	Yes	Yes	Yes	Yes	No
20	29	F	CD	Yes	No	No	Yes	Yes	No

*CD*, Cushing’ s disease; *CSA*, cortisol-secreting adenoma; *IGT*, impaired glucose tolerance.

Active disease status was defined as the concurrent presence of a mean urinary free cortisol (mUFC) level > 110 μg/24 h and at least one of the following: DST 1 mg > 1.8 μg/dL, late night salivary cortisol (LNSC) > 0.30 μg/dL, recurrence of clinical signs of hypercortisolism, or worsening of any existing ones, in the absence of GC therapy. Subjects under medical treatment were also considered to have active disease, which was classified as controlled or uncontrolled based on mUFC levels (≤ or > ULN). Additionally, 14 age and sex-matched healthy volunteers (3 males and 11 females, average age 50 ± 16 years) were selected as control group (CTRL). The study was conducted in accordance with the Declaration of Helsinki. Signed informed consent for molecular analysis of blood samples and for access to clinical data was obtained from all patients, and the study was approved by Polytechnic Marche University Ethical Committee.

### Two-Step Isolation of Neuron-Derived Exosomes (NDEs)

Neuron-derived exosomes (NDEs) were isolated from 0.75 mL of frozen human plasma containing EDTA. Samples were defibrinated with thrombin (System Biosciences, Inc., Mountainview, CA) by a 30-min incubation at room temperature. After the addition of PBS, samples were mixed, left for 5 min at RT and then centrifuged at 4000 × *g* for 20 min. Supernatants were transferred to fresh tubes and gently mixed after addition of ExoQuick exosome solution (System Biosciences, Inc., Mountainview, CA). Suspensions with ExoQuick were incubated for 60 min at 4 °C to precipitate total exosomes [36] and then centrifuged at 1500 × *g* for 30 min at RT. Supernatants were discarded after centrifugation and the pellet containing exosomes was re-suspended in 0.5 mL of PBS.

Previous studies indicated that neuronally derived exosomes can be detected in plasma using L1 cell adhesion molecule (L1CAM-CD171), a surface protein located on neurons, and that their contents reflect protein expression in the brain [37, 38]. Therefore, to enrich for exosomes containing L1CAM, suspensions were incubated overnight with 4 µg of mouse anti-human CD171 (clone 5G3, eBioscience, San Diego, CA) in a total volume of 50 µL of 3% BSA for tubing in agitation on wheel. Subsequently, 15 µL of streptavidin-agarose Ultralink resin (Thermo Scientific, Rockford, IL, USA) were added to 25 µL of 3% BSA and incubated for 30 min at 4 °C with continuous mixing. After centrifugation at 200 × *g* for 10 min at 4 °C and removal of the supernatant, each pellet was re-suspended in IgG elution solution (Pierce ™ IgG Elution Buffer, Thermofisher Scientific) by mixing and later centrifuged to detach L1CAM + Exosomes from the bead-antibody complex. After adding 15 µL of pH 8 Tris–HCl to neutralize the solution, the final suspensions containing neuronal exosomes were kept at − 80 °C.

### Immunoblotting of NDEs

Protein-based quantitation of isolated exosomes was done using the Pierce BCA Protein assay kit (Thermofisher Scientific) as reported by the manufacturer. Subsequently, an equal amount of neuronal exosomes (17.6 µg of total protein for L1CAM and 5 µg for CD81), resuspended in 1:40 of βME, 1:4 of Laemmli buffer, and RIPA buffer, was denatured by sonication with 10 pulses at 600 W, boiled at 95 °C for 10 min and then resolved on 12% Tris–glycine gel precast for electrophoresis (12% Mini- PROTEAN® TGX ™ Precast Protein Gels). Resolved proteins were transferred in duplicate onto nitrocellulose or PVDF membrane: one membrane was used to verify the transfer by Ponceau Red staining; the other was used for immunoblotting and blocked by incubating in 5% TBS-BSA to minimize non-specific binding of antibodies. Blocked blots were submerged with primary antibodies diluted in 5% BSA in TBS at the following concentrations 0.5 μg/mL CD171 (Invitrogen Catalog # 13–1719-82) or 1:250 CD81 (Invitrogen Catalog # MA5-13,548) overnight at 4 °C in agitation and subsequently washed three times (10 min each) with 1X Tris buffer saline (TBS) buffer followed by incubation with 5% BSA in TBS with 0.5% Tween-20 containing the goat-anti mouse secondary antibody conjugated with horseradish peroxidase (1:20,000 Chemicon International, Catalog # AP124P). Unbound antibodies were removed by washing with 1X TTBS buffer (3 × 5 min), and signal was recorded using Clarity Western ECL Substrate kit under Bio-Rad ChemiDoc Imager (Hercules, CA, USA).

### Specimen Preparation for Scanning Electron Microscopy (SEM)

The purified neuronal exosomes were then reprecipitated for scanning electron microscopy analysis. The samples were suspended in PBS and centrifuged for 2 h at 100,000 × *g* at 4 °C in an ultracentrifuge. The exosome pellet was fixed with 2.5% glutaraldehyde in 0.1 M sodium phosphate solution (pH 7.2) for 1 h at 4 °C. Then, the excess of fixative was removed, and the pellet rinsed with the same buffer.

For SEM observation, a 5 µL drop of fixed exosome pellet was put on the surface of coverslip previously covered with poly- L-lysine, by immersing it directly in a solution of 0.1% poly-L-lysine hydrobromide (MW 85,000) in distilled water and allow to dry for few hours at room temperature. The coverslip was incubated overnight at 4 °C in a moist chamber for 24 h and then washed with 0.1 M phosphate buffer to remove poly-L-lysine, post fixed in 1% OsO 4 in 0.1 M phosphate buffer for 1 h at RT, then quickly washed in the same buffer and dehydrated in a graded alcohol series until 100%. After chemical fixation, post-fixation, and ethanol dehydration, the specimen was critical point-dried using CO2, mounted on aluminum stubs, sputter coated with gold palladium and finally observed with a FEI Quanta 200 FEG environmental scanning electron microscope.

### Real-time PCR (RT-qPCR)

The analysis was performed on neuronal exosomes isolated from plasma of CS patients and healthy controls. The RT-qPCR protocol includes the isolation of total RNA, performed through the Total RNA isolation kit (Norgen Biotek, Thorold, ON, USA) following the manufacturer’s instructions. The concentration and purity of RNA were determined using a NanoDrop ND 2000 spectrophotometer (Thermo Scientific, San Jose, CA, USA). RNA was stored at − 80 °C until use. For analysis, the human miRNAs (miR-126, miR-9, miR-223, miR-34a, miR-124a, and miR-146a) were quantified by RT-qPCR using TaqMan MicroRNA assay (Applied Biosystems, Foster City, CA, USA) according to the manufacturer’s guidelines. RT-qPCR data were standardized using the cell-miR-39 as spike in. The specificity of the product was examined by dissociation curve analysis. The results were calculated using the delta Ct method for analyzing the modulations with respect to the spike-in according to the 2-∆Ct formula (the relative expression indicated in the figure are normalized to cel-miR-39 spike-in).

### Bioinformatic Analysis of miRNA Target Genes Implicated in Adult Neurogenesis

The bioinformatic analysis of the correlation between the investigated miRNAs and the targets was carried out using three databases for the correlation miRNA-target gene (TargerScan 8.0 [39], miRBD [40], HMDD v3.2 [41]), considering only the genes shared by both databases and with a prediction score above 0.7. Their function was further examined using the GeneCards database [42] to see whether the target genes were involved in adult neurogenesis. Finally, the function described in the database was validated by literature (see Table 1) to identify only the major genes involved in the adult neurogenesis process. In addition, using the TAM 2.0 database [43], a Gene Ontology (GO) study was carried out to link the deregulated miRNAs with both their function in cellular activity and their correlation with the primary symptoms observed in Cushing’s syndrome patients.

### Statistical Analysis

Data are expressed as mean ± SEM (standard error of the mean). The number of samples (*n*) in each experimental condition is reported in figure legends. Data was analyzed using the commercial program GraphPad PRISM 6.0 (GraphPad Software, USA). The comparison of continuous variables between two groups was performed by the Mann–Whitney Test.

## Results

### Neuronal Exosome Authentication

**Fig. 1:**
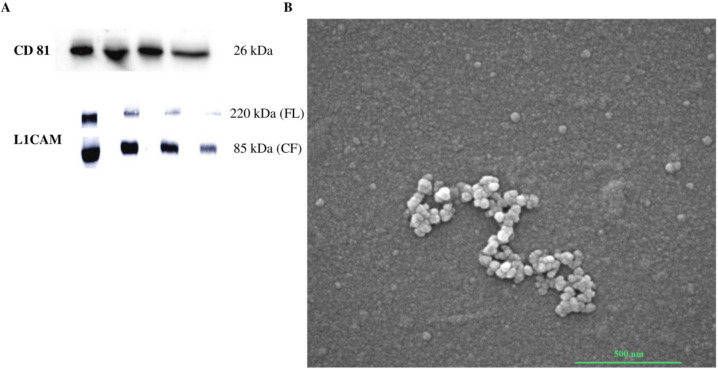
Authentication of plasma neuronal extracellular vesicles. **A** Representative western blot performed on CTRL exosomal samples showing classical marker CD81 and neuronal exosomal specific marker L1CAM that was enriched with anti-L1CAM capture (FL- full length CL-cleaved form). **B** Representative scansion electron micrographs showing the typical morphology and dimension of anti-L1CAM-captured neuronal exosomes

A validated protocol for isolating neuronal exosomes from human plasma has been used [44, 45]. According to the literature [46], our isolated vesicles were positive for CD81, a classic exosomal marker, and enriched for the neuronal exosome-specific marker L1CAM (Fig. 1A). L1CAM was present in both cleaved and full-length isoforms (Fig. 1A) [46]. Scanning electron microscopy analysis was also performed to characterize the morphology of plasma neuronal exosomes derived from healthy subject NDE pool. As shown in Fig. 1B, all NDEs had a vesicular shape with diameters ranging from 50 to 150 nm (Fig. 1B) consistent with exosome size.

### NDE miRNA Content Analysis

**Fig. 2:**
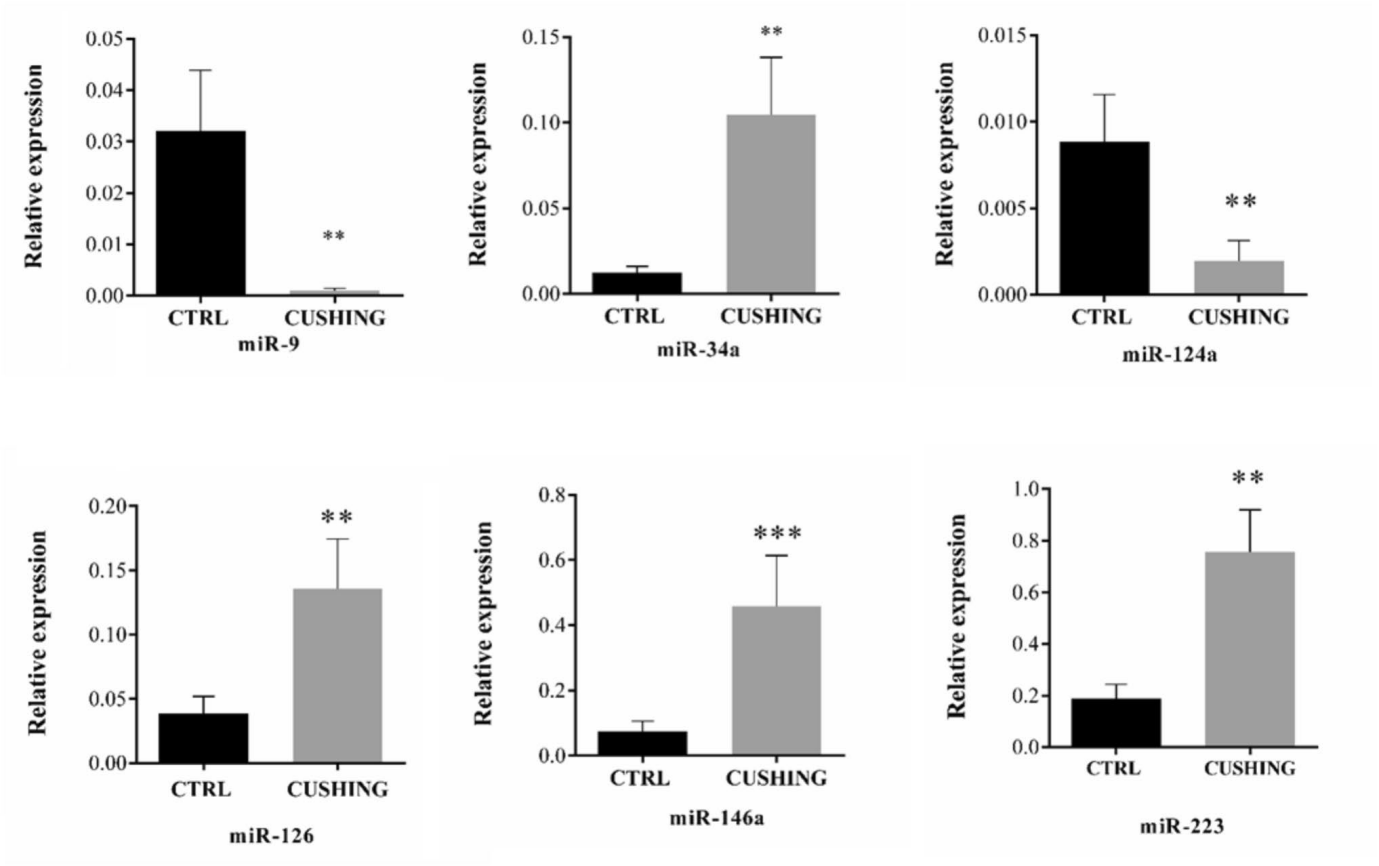
Quantitative reverse-transcription polymerase chain reaction (RT-qPCR) of neurogenesis-related miRNAs in neuronal exosomes. The data were normalized to cel-miR-39 spike-in. Number of samples: miR-9 CTRL (8) CUSHING (8); miR-34a CTRL (10) CUSHING (8); miR-124a CTRL (12) CUSHING (12); miR-126 CTRL (15) CUSHING (9); miR-146a CTRL (15) CUSHING (11); miR-223 CTRL (14) CUSHING (11). Mann–Whitney test was performed: *p < 0.05; **p < 0.01; ***p < 0.001

Based on the literature, we selected 6 miRNAs (hsa-miR-9, hsa-miR-34a, hsa-miR-124a, hsa-miR-146a, hsa-miR-223, and hsa-miR-126) that are involved in neuroplasticity processes, such as adult neurogenesis [47–52] to evaluate by qRT-PCR their levels in NDEs. The qRT-PCR results revealed that all the miRNAs analyzed were significantly differentially expressed in CS patients (CUSHING) as compared to healthy control subjects (CTRL) (Fig. 2). In particular, miR-9 and miR-124a were down-regulated, while miR34a, miR-126, miR-146a, and miR-223 were up-regulated in CS patients (Fig. 2).

### In Silico Analysis of miRNAs Target Genes

**Fig. 3:**
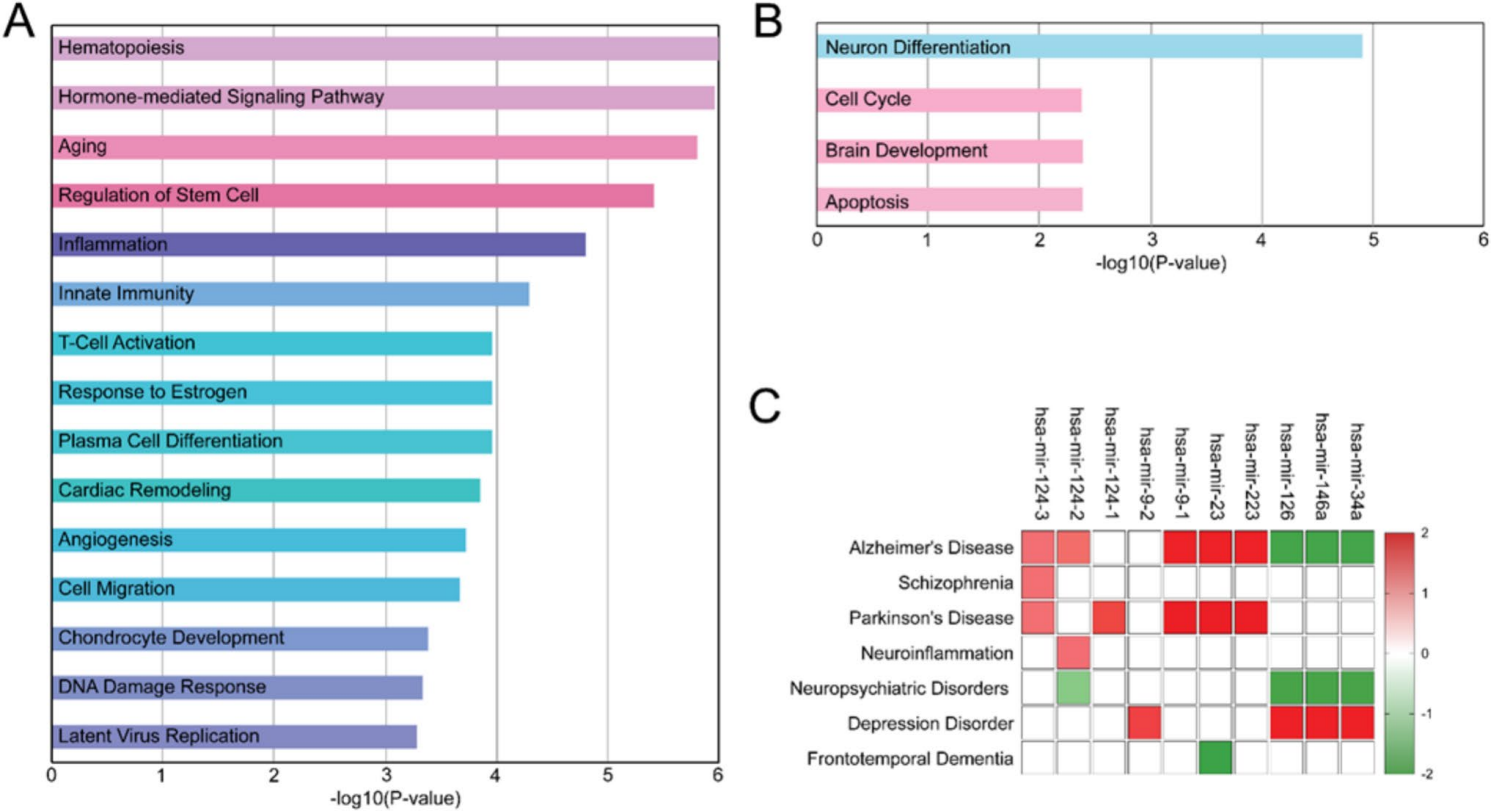
In silico functional analysis. **A** Gene Ontology for cellular functions of up-regulated miRNA. The cellular processes reported are significant in their enrichment (*p* < 0.05). **B** Gene Ontology for cellular functions of down-regulated miRNA. The cellular processes reported are significant in their downregulation (*p* < 0.05). **C** Heatmap of a comprehensive perspective of miRNA deregulation in CS. The palette is related to the association score. Green boxes represent down-regulated miRNAs, whereas red boxes represent up-regulated miRNAs

We performed functional annotation analysis (Gene Ontology) of genes that were targeted by these deregulated miRNAs. Functions were differentially enriched, including regulation of stem cells, cell migration, hormone mediated signaling response for up-regulated miRNAs (Fig. 3A), while neuronal differentiation, cell cycle regulation, and brain development for down-regulated miRNAs (Fig. 3B). Furthermore, CS patients exhibited neurological/psychiatric pathological conditions, hypertension, cardiovascular disease, coagulopathy, diabetes, dyslipidemia, obesity, hypokalaemia, and osteoporosis. Considering this evidence, a correlation study was performed, allowing the up and down regulation of the studied miRNAs to be correlated with any comorbidities. There are correlations between the symptoms, such as depression, schizophrenia, neuroinflammation and neuropsychiatric disorders, and the dysregulation of miRNA levels found within extracellular vesicles of neuronal origin, as shown in Fig. 3C.

Subsequently, we conducted an analysis aimed at establishing a correlation between the activity of dysregulated miRNAs and their corresponding target genes. Three distinct databases, namely, miRDB, TargetScan 8.0, and HMDD v3.2, were utilized for this purpose. Common target genes identified across all databases were subjected to further analysis via the GeneCards database, which facilitates the determination of a gene’s function. If the function of a gene included processes such as neural differentiation, neurogenesis, or cell cycle regulation, additional literature investigation was conducted to confirm its involvement in adult neurogenesis.

In Table 1 are listed the genes that exert the most significant influence on the neurogenesis process regulated by the examined miRNAs. Notably, most of these genes are part of the Wnt and Notch signaling pathways, which are central regulators of adult neurogenesis across all levels [53, 54]. Thus, these findings can underpin the hypothetical correlation between miRNA levels and adult neurogenesis impairment.

The relationship between dysregulated miRNA cargo in CS patient’ exosomes and their target genes differs depending on the miRNA under investigation. For example, miR-9 and miR-124a, down-regulated if compared to controls, resulting in increased expression of TLX, SOX2, FOXG1, HES1, MEF2 C, SOX9, SIRT1, NEUROD1, PTBP1, LHX2, REST, BDNF, and DCX genes. MiR-126, miR-223, miR-146a, and miR-34a, on the other hand, are up-regulated, resulting in down-regulation of their target genes (Table 2). Although some genes may overlap, the observed variation in miRNA levels has been widely linked in the literature to alterations in adult neurogenesis [47–52, 55].

**Table 2:**
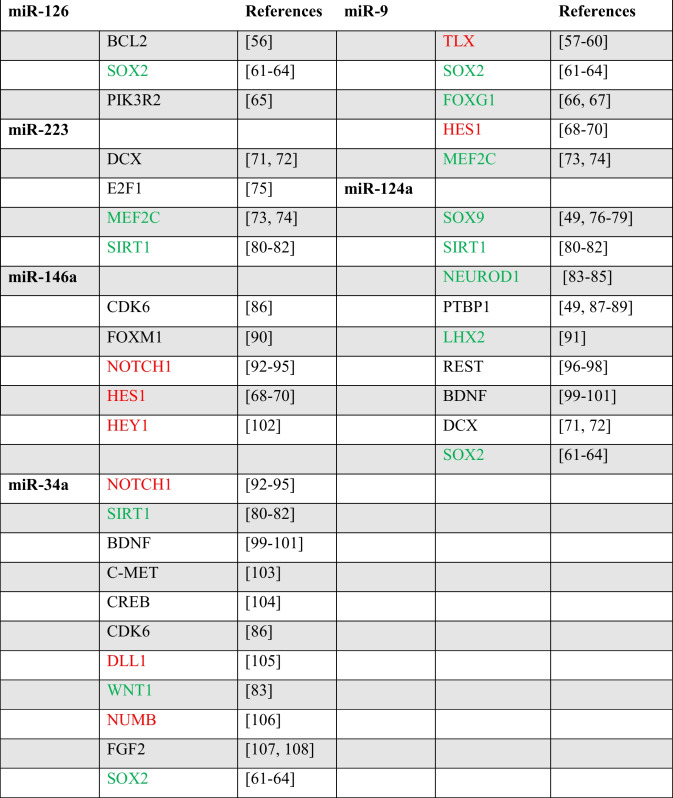
Gene target of up- and down-regulated miRNAs involved in adult neurogenesis (green: Wnt pathway associated genes; red: Notch pathway associated genes)

## Discussion

The current exploratory study aimed to acquire insight into the role of neuronal-derived exosome cargo as a potential circulating biomarker to in vivo monitor neuroplasticity processes in healthy and diseased adult humans. As a matter of fact, exosome’s contents likely reflect expression of various biomarkers in brain tissues, such as neurogenesis-related miRNAs, which can be altered and dysregulated in pathological states. To achieve this purpose, we used peripheral blood of healthy subjects and patients affected by CS, a condition characterized by chronically elevate glucocorticoids circulating and tissutal levels known to have a negative impact on adult neurogenesis from a wealth of evidence obtained on animal models [109, 110]. The main findings of the research are (i) neuronal-derived exosomes were identified in the plasma of both adult healthy volunteers and in patients with endogenous CS; (ii) miRNA cargoes were quantified in neuronal exosomes derived from both experimental groups; (iii) the neuroplasticity-related miRNAs evaluated in this study were differently expressed in CS patients as compared to healthy ones; (iv) the different expression of the selected miRNAs may underlie the deficit in cognitive and affective function observed in CS patients, suggesting these miRNAs as diagnostic biomarkers in the initial phase of the disease and/or in mild CS lacking the classic detectable manifestations of overt CS.

The miRNAs we selected (hsa-miR-9, hsa-miR-34a, hsa-miR-124a, hsa-miR-146a, hsa-miR-223, and hsa-miR-126) are involved in a range of biological processes, including various aspects of adult neurogenesis such as NSPC proliferation, apoptosis, differentiation, and neuronal maturation [111]. In this regard, considering the biological activities of the selected miRNAs, miR-9 is known to play a key role in regulating neural progenitor behavior and promoting neuronal differentiation [112]. Consistently, the expression of miR-9 in human fibroblasts, in synergy with miR-124, is sufficient to convert them into neurons [113]. Here, we found out that miR-9 expression is significantly down-regulated in circulating neuronal exosomes of CS patients compared to healthy subjects, thus indicating a reduced neuronal differentiation under disease conditions. An impairment in neuronal differentiation may result in a decrease of new neuron production in the neurogenic brain region, such as hippocampus, and in associated cognitive and affective dysfunction [114–116]. Consistently, blocking miR-9 in the hippocampus evoked deficits in learning and memory in rodents [112].

miR-124 is the other miRNA we found down-regulated in CS patients like miR-9. miR-124 is the most brain-rich miRNA whose main function is to restrain NSPC proliferation and to promote neuron differentiation by targeting several genes among which Sox9. Suppression of Sox9 mediated by miR-124 is necessary for neuron formation [117]. More importantly, proper neuronal differentiation of progenitor cells may require narrow limits of GCs and miR-124 was identified as regulator of cell responsiveness to GCs by reducing GR protein levels in neuronal tissues [118]. Thus, in patients affected by CS, miR-124 downregulation may fail to reduce GR protein levels, possibly inducing an increase of brain cell responsiveness in condition of high GCs exposure. Since GC receptors are expressed in neuronal progenitors and immature neurons [119], miR-124 reduced expression makes them particularly vulnerable to GCs effects, impairing neurogenesis in CS patients.

miR-34a is also involved in adult neurogenesis affecting neuronal differentiation mainly through its multiple targets within the Notch signaling pathway [120]. Moreover, an aberrant increase or decrease in its expression causes apoptosis of progenitor cells [121]. Our findings showed that in CS patients miR-34a is overexpressed in circulating neuronal exosomes, suggesting an impairment in newborn cell survival and neuronal differentiation.

Neuronal differentiation can be regulated by miR-223 that seems to be negatively correlated with neurogenesis: its increased expression inhibits cell migration by regulation of genes related to the cytoskeleton, and immature neuron differentiation [122]. We detected in neuronal-derived exosomes isolated from the blood of CS patients an overexpression of miR-223, suggesting detrimental neuronal differentiation and migration.

miR-146a is an additional microRNA that is upregulated in CS patients. Its overexpression has been found in mouse/rat models of neurological diseases, such as depression, epilepsy, traumatic brain injury, and Alzheimer disease [50, 55]. The increase of miR-146 expression is related to reduced neurogenesis, by decreasing newborn cell survival [123].

Regarding the miR-126, evidence suggests that it is positively related to adult neurogenesis, by promoting the proliferation and survival of neural stem cells [124]. However, its overexpression has been shown to be neurotoxic by impairing IGF/PI3 K/AKT signaling, whereas its inhibition is neuroprotective [125], suggesting that miR-126 could be involved in the general survival mechanisms of neurons. It deserves to be mentioned that PI3 K/AKT signaling is involved in psychiatric disorders, such as depression, and neurogenesis is negatively associated with depression [126]. Long-term exposure to hypercortisolism, as in Cushing’s disease, negatively affects mental health of patients, frequently inducing depression [126].

Furthermore, proneural factors, such as NeuroD, regulated by the investigated microRNAs, control multiple stages of neurogenesis, from neural progenitor cell specification to the development of a pan-neuronal phenotype [85]. Additionally, molecules related to adult neurogenesis, such as BDNF and REST, play complex roles in promoting the generation, survival, differentiation, maturation, and integration of new neurons into existing brain circuits [96, 97, 100]. CREB phosphorylation has been observed in newly generated immature neurons in the subgranular zone (SGZ) of the hippocampus [127]. pCREB is consistently present in newly formed granule cells in the hippocampus, and its expression correlates with the neuronal marker doublecortin, which decreases as neurons transition from immature to mature stages defined by calbindin expression [128]. Furthermore, Cdk6 is essential for cell proliferation in the dentate gyrus of the hippocampus and the subventricular zone of the lateral ventricle. Specifically, Cdk6 deficiency restricts the expansion of neuronally committed precursors by prolonging the G1 phase, thereby limiting the generation of new neurons [86].

Altogether, these findings obtained from circulating neuronal exosome miRNA analysis could support that adult neurogenesis may be impaired in CS, probably due to prolonged exposure to high levels of cortisol, as occur in animal models. Consistently, the functional network analysis of miRNA targeted genes we performed shows that there is a correlation between the deregulation of the examined miRNAs and the adult neurogenesis process that involves signaling pathways regulating pluripotency of stem cells, cell migration and neuronal differentiation (Fig. 3A, B). In addition, the up and down regulation of the considered miRNAs correlates with comorbidities frequently exhibited by CS patients (Fig. 3C). In fact, chronic GC exposure determines many complications, including obesity, insulin resistance with glucose intolerance and diabetes, dyslipidemia, hypertension, thromboembolism, neuropsychiatric disorders, and susceptibility to infections and sepsis. Glucocorticoid excess can contribute, directly or indirectly through these conditions, and the drugs used to treat them, to changing miRNAs. Moreover, amenorrhea, the absence of menstruation, observed in female CS patients, can have complex effects on the brain, particularly on neurogenesis. Indeed, hormones such as estrogen and progesterone, which are typically disrupted in amenorrhea, play crucial roles in brain health and the formation of new neurons, especially in the hippocampus. In turn, dysregulation of specific miRNAs can contribute to both the causes and consequences of amenorrhea, including its impact on reproductive health, metabolism, and the brain.

In conclusion, our exploratory study demonstrates that the selected miRNAs related to neuroplasticity processes—such as neurogenesis—and enriched in circulating neuronal-derived exosomes are dysregulated in patients with CS. Overall, this dysregulation may suggest a likely reduction in new neuron production in neurogenic brain regions, such as the hippocampus, which may partly account for the early cognitive and affective deficits observed in CS patients. Consistently, several studies have shown that the dysregulation of the miRNAs we selected may mechanistically contribute to the well-documented mood and memory disturbances associated with prolonged hypercortisolism [129–133]. To our knowledge, only a few studies have investigated circulating miRNAs in CS [134] and none have specifically analyzed those contained in neuronal-derived exosomes, which may serve as early indicators of neurological dysfunction—even before the overt manifestation of clinical symptoms. Circulating miRNAs represent minimally invasive diagnostic markers and may prove useful for the early detection of CS, enabling prompt intervention, particularly in the initial stages or in cases of mild CS. Normalization of cortisol levels could potentially halt or prevent the progression of hippocampal-related impairments, including memory and affective dysfunction.

However, our study is not without limitations. The primary limitation was the relatively small sample size. Although this limitation exists, the number of CS patients included in our study was sufficient to detect significant findings in a very rare disorder, with an estimated incidence ranging from 1.8 to 4.5 cases per million people per year, and a prevalence of 57–79 cases per million. The second limitation concerns the miRNAs analyzed in our study, as they have been associated with multiple neurological disorders such as Alzheimer’s disease, epilepsy, and traumatic brain injury (TBI). However, the enrolled CS patients did not suffer from any of these conditions. The third limitation involved relating differences in miRNA expression levels to neurogenic changes occurring in a specific subregion of the hippocampus, considering that the exosomes originated from the whole brain. Nevertheless, the hippocampus is particularly sensitive to glucocorticoid regulation due to its high expression of glucocorticoid and mineralocorticoid receptors [134]. Chronic stress and pathologically elevated glucocorticoid levels, as observed in Cushing’s syndrome, have been shown to markedly reduce hippocampal neurogenesis, impair synaptic plasticity, and contribute to mood and cognitive dysfunction [15, 135, 19]. However, given the expression of the selected miRNAs also in other brain regions and their involvement in multiple neurological and systemic conditions, the observed changes could also reflect broader effects of Cushing’s disease.

Ultimately, when considering the results as a whole and in light of the limitations, our exploratory study suggests that the identification of specific neuronal exosomal cargoes could serve as a potential biomarker for monitoring both functional and dysfunctional neuroplasticity processes in adult humans. Moreover, in line with previous studies [136], it supports the need to discover new biomarkers for the diagnosis and prevention of neurological diseases.

## Data Availability

No datasets were generated or analysed during the current study.
